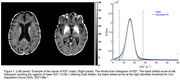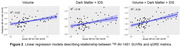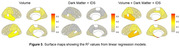# Relationship Between 18F‐AV‐1451 Binding in Human Brain and Tissue Alterations Defined by Quantitative Gradient Recalled Echo (qGRE) MRI Measurements

**DOI:** 10.1002/alz.083829

**Published:** 2025-01-09

**Authors:** Satya V.V.N. Kothapalli, Tammie L.S. Benzinger, Brian A. Gordon, Cihat Eldeniz, Charles F Hildebolt, Manu S. Goyal, John C. Morris, Dmitriy A. Yablonskiy

**Affiliations:** ^1^ Washington University School of Medicine in St. Louis, St. Louis, MO USA; ^2^ Hope Center for Neurological Disorders, Washington University in St. Louis, St. Louis, MO USA; ^3^ Knight Alzheimer Disease Research Center, St. Louis, MO USA; ^4^ Washington University in St. Louis School of Medicine, St. Louis, MO USA

## Abstract

**Background:**

The 18F‐AV‐1451 radioligand enables in‐vivo identification of tau neurofibrillary tangles that are considered as biomarkers of neurodegeneration in Alzheimer Disease (AD). However, off‐target radioligand binding is also observed in basal ganglia, known as an iron‐rich region. Hence, it is important to distinguish between radioligand‐identified tissue neurodegeneration and iron‐related radioligand binding effects. Our aim is to answer this question by using previously developed quantitative Gradient Recalled Echo (qGRE) MRI technique sensitive to morphological and microstructural tissue neurodegeneration, as well as to iron deposition.

**Methods:**

Sixty‐eight participants (ages: 72.1y ± 8.2y; 15 with amyloid status (Aβ) positive and cognitive dementia rating (CDR®)>0; 16 with Aβ‐positive and CDR®=0; and 37 with Aβ‐negative and CDR®=0) recruited by Knight ADRC underwent 18F‐AV‐1451 PET imaging and qGRE MRI imaging (3T Siemens MRI scanners). As previously demonstrated, the R2t* metric of qGRE signal is sensitive to both, pre‐atrophic neuronal loss (low R2t*, a.k.a. Dark Matter (DM), and nonheme iron accumulation (High R2t*). The histogram characterizing distribution of R2t* values across the brain (Figure 1) allows the quantification of DM (R2t*<5.8s^‐1^) and Iron Deposition Score (IDS, R2t*>28s^‐1^). 18F‐AV‐1451 binding was characterized in terms of Standardized Uptake Value Ratio (SUVR). All results were summarized in brain regions segmented using FreeSurfer.

**Results:**

(Figures 2 and 3): Tissue volumes exhibited moderate negative associations with ^18^F‐AV‐1451 SUVRs in MTL (R^2^=0.21), temporal lobe (R^2^=0.17), and parietal lobe (R^2^=0.11). The linear model that includes IDS and DM exhibited positive association with ^18^F‐AV‐1451 SUVRs only in MTL (R^2^=0.18). The linear mixed model that includes volume, IDS and DM metrics showed the improved association between predicted and actual 18F‐AV‐1451 tracer retention in the MTL (R^2^=0.34), the temporal lobe (R^2^=0.23), and the parietal lobe (R^2^=0.12).

**Conclusion:**

Our results suggest that the ^18^F‐AV‐1451 binding is modulated not only by neurodegeneration but also by elevated levels of iron deposition. More detail studies are required to answer the question whether the iron effect is concomitant or contributes to neurodegeneration through ferroptosis. The qGRE‐based multiparametric approach allowing simultaneous identification of pre‐atrophic neurodegeneration and iron deposition has a potential to contribute to understanding of this intricate relationship.